# Influence of cold atmospheric pressure plasma treatment on germination and plant biomass of *Trifolium pratense* L.

**DOI:** 10.1371/journal.pone.0332166

**Published:** 2025-09-09

**Authors:** Mareike Kavka, Henrike Brust, Christine Brandt, Thalita M. C. Nishime, Evelin Willner, Nicola Wannicke, Klaus J. Dehmer

**Affiliations:** 1 Satellite Collections North, Genebank Department, Leibniz Institute of Plant Genetics and Crop Plant Research (IPK), Malchow/Poel, Germany; 2 Department Plasma Bioengineering, Leibniz Institute for Plasma Science and Technology (INP), Greifswald, Germany; 3 Department Plasma Sources, Leibniz Institute for Plasma Science and Technology (INP), Greifswald, Germany; Shahrekord University, IRAN, ISLAMIC REPUBLIC OF

## Abstract

Treatment of seeds with cold atmospheric pressure plasma (CAPP) is in its proof-of-concept phase with regard to its effect on germination and plant growth. To increase the germination of hardseeded red clover (*Trifolium pratense* L.), seeds are usually scarified, which is time-consuming and labour-intensive. The aim of this study was to compare the effect of different CAPP devices (indirect treatment: plasma processed air, direct treatment: corona discharge, argon and air dielectric barrier discharge) on germination and early growth of different long-term stored red clover accessions and to determine whether germination can be increased to meet seed management requirements. Sixty different red clover seed lots (diverse accessions and harvest years) with different initial germination percentages were divided into three batches of 20 lots each and the effect of the different plasma treatments on germination and development were examined in laboratory and greenhouse. The overall results indicate a plasma discharge- and accession-depended enhancement of germination speed which was detected in all batches but most pronounced in Batch 1. While direct treatments, especially with corona discharge-plasma, increased germination speed (up to 58% germination seven days after sowing vs. 44% in control in laboratory conditions), treatment with plasma processed air resulted partially in reduced germination speed (42%). Despite a small but significant increase in total germination of maximum five percentage points, no treatment led to an increase from 62% or 70% in control (depending on experiment) to at least 80% germination percentage to meet storage requirements for seed banks. Stimulating effects on biomass of young plants under greenhouse cultivation conditions were observed in Batch 1, but were absent in Batch 2 and 3 and therefore inconclusive. Future research is needed to elucidate influencing factors on plasma effects in red clover seed lots which include but are not limited to the effect of seed coat compounds and seed coat thickness.

## Introduction

Red clover (*Trifolium pratense* L.) has a wide geographical and climatic spread [1] and is one of the world’s most important perennial forage legumes [2,3], used for silage and hay [1]. Due to the ability to fix atmospheric nitrogen (N_2_) via symbiotic rhizobial bacteria, red clover plays an important role in sustainable agriculture as it can increase nitrogen (N) levels in the soil and reduces therewith the demand of following crops for N fertilizers [4,5].

Physical dormancy because of a hard seed coat is common in legumes [6] and may lead to low germination rates. Natural hardseededness is a mechanism of seed dormancy that determines germination depending on various environmental conditions. Under natural environmental conditions, the seed coat becomes permeable by the action of abiotic factors like temperature, moisture or light [6,7]. Thereby, the level of hardseededness as a result of genetic and environmental influences differs not only between species, but also between different populations and even between harvest years [6,8]. Low germination rates are of significant concern especially for long-term seed preservation by gene banks, for seed producers and breeders. Mechanical etching with quartz sand – scarification – is a conventional pre-treatment of red clover seeds to increase germination. Apart from mechanical scarification, acidic scarification with concentrated sulfuric acid, pre-heating or pre-cooling as well as soaking in potassium nitrate are methods to increase germination in red clover [9,10]. However, all are time-consuming and labour-intensive. In general, modern biological and chemical seed treatment methods are applied to ensure uniform germination and improve seedling emergence and vigour. These methods encompass biological seed coating with beneficial microbes such as *Trichoderma* and *Bacillus* species to protect seeds from soil pathogens, promote nutrient uptake and enhance germination and seedling vigour [e.g., 11 and references therein]. Moreover, hormonal and chemical priming is applied using, e.g., kinetin, gibberellic acid (GA_3_) [e.g., 12], indole-3-acetic acid (IAA) or melatonin [e.g., 13]. Chemical treatment covers also the fast-growing division of nanotechnology [14] which includes application of nanofertilisers and nanopesticides. One of these applications is the usage of inorganic nanoparticles like selenium and zinc oxide for priming [15,16]. Apart from this, physical methods are adopted [17], including ultrasound [18], magnetic field treatment [19], irradiation [20], and thermal stratification (especially for agroforestry).

An alternative physical seed treatment technology in its proof-of-concept phase is cold atmospheric pressure plasma (CAPP). Plasma is the fourth state of matter and contains, among others, charged particles, reactive oxygen and nitrogen species (RONS), excited molecules and UV photons [21,22]. CAPP can be generated by supplying energy to a neutral gas at atmospheric pressure causing the formation of charge carriers [23]. This energy can be supplied as thermal energy, but often electrical energy is used. Reactor configurations [24] range from dielectric barrier discharges (DBDs), plasma jets, spark discharges, corona discharges (CDs) to microwave discharges. The resulting different kinds of discharges can deviate in the qualitative and quantitative composition of above-mentioned components, depending on the plasma device geometry, applied voltage, current, frequency and feeding gas used (e.g., air, helium, argon, nitrogen, oxygen). The mode of CAPP exposure to the target can be direct or indirect via the generation of plasma treated gas (e.g., plasma processed air, PPA) or liquids (e.g., plasma processed water) [21,25,26]. Especially in direct treatment modes, electrical fields and temperature of the gas discharge further influence the impact on biological targets [27,28].

CAPP is capable of eliminating pathogenic microorganisms from the seed surface [29], but also of promoting seed germination and seed viability [30–32]. Most published studies indicate increased germination rates after CAPP treatment, including enhanced seedling growth and/or changes in secondary metabolites, e.g., after seed treatment of red clover [33–35] and of other plants including further legumes [31,36–38]. In the studies with red clover [33–35], the plasma device was a planar geometry reactor and plasma was ignited at a pressure of 200 Pa. Seeds were counted as germinated when 1 mm of the radicle emerged. This increase was mainly associated with changes in physical and chemical properties of the seed surface, such as increased hydrophilicity and water permeability after CAPP treatment [38,39]. A physiological result of CAPP treatment that might lead to increased germination is the formation of a wide range of RONS acting as signalling molecules during the germination process [40–42].

So far, studies that consider a large number of accessions and seed lots of a plant species in combination with different types of plasma exposure are lacking. Thus, the aim of our study was to compare the effect of different CAPP treatment systems on germination and early growth of different long-term stored red clover accessions of the German Federal *Ex situ* Gene Bank hosted by the Leibniz Institute of Plant Genetics and Crop Plant Research (IPK; [43]) with a wide range of initial germination rates. In total, we assessed the effect of plasma treatments (different kinds of plasma discharge configurations; indirect treatment: plasma processed air, direct treatment: corona discharge, argon and air dielectric barrier discharge) on the germination of 60 different seed lots from different origins and harvest years to identify an easy-to-use physical treatment method for increasing the germination percentage of red clover gene bank material and thus – in the best-case scenario – to meet storage requirements not achieved untreated.

## Materials and methods

### Plant material

A set of 38 accessions of red clover was selected from the IPK’s oilseed and forage crops collection (S1 Table in S2 File). The set comprised diploid as well as tetraploid accessions from at least 23 different countries in Europe, North America and Asia (S1 Table in S2 File). Twenty-two of the accessions were tested with seed lots from two different harvest years and 16 accessions were tested with one seed lot, resulting in 60 different seed lots. The harvest years of the seed lots ranged from 1975 to 2020 (S2 Table in S2 File). Seeds were stored at −5 °C with 5–8% water content. These seed lots were chosen because of their different initial germination capacities in the last routine germination test, ranging from low (<50%) to medium (50–69%) and high (>69%). Due to the extensive and time-consuming investigations, the set was divided into three batches (Batch 1, 2, 3), which were treated and tested separately (S2-S4 Tables in S2 File).

### Seed treatment with atmospheric pressure plasma

**Fig 1:**
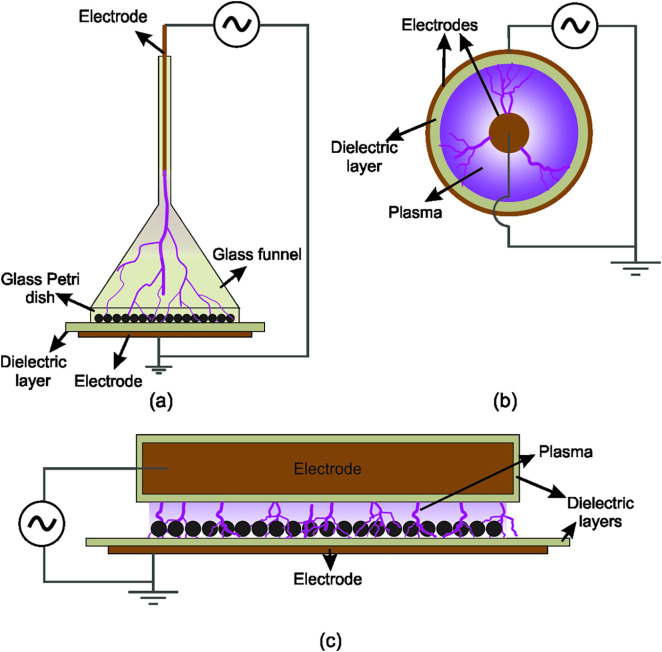
Electrode arrangements for the direct treatment reactors. (a) corona-discharge reactor (CD; adapted from [44]), (b) dielectric barrier discharge in argon (ArDBD; adapted from [45]) and (c) dielectric barrier discharge in air (airDBD).

Four different plasma devices were used and compared to an untreated control. Non-scarified seeds were treated with plasma either directly or indirectly. For the direct treatment, three different reactor configurations were used (Fig 1, S5 Fig in S1 File). These were a conical CD reactor, a coaxial DBD reactor with argon as working gas (ArDBD), and a DBD in air (airDBD). Individual plasma treatment parameters were narrowed down to a total of five parameters during pre-tests to prevent using unfavourable parameters for seed germination and due to restrictions in the amount of seeds within some lots. The air humidity was kept constant at around 30% for all direct plasma treatments.

#### Corona discharge (CD) reactor.

The CD reactor had a conical shape consisting of an upside-down funnel mounted on top of a glass Petri dish (80 mm in diameter) for treatment of small seeds (Fig 1A). At the funnel axis, a tungsten rod served as the high voltage electrode. The grounded electrode was placed below the Petri dish covered by a glass dielectric layer. The reactor, fed with 4 slm (standard litres per minute) of argon, generates a filamentary discharge that extends from the pin electrode to the Petri dish surface (S5 Fig in S1 File). Details of this reactor configuration are reported in [44]. The CD reactor was operated at a frequency of 10 kHz and applied voltage of 24 kV p-p with voltage duty cycle of 50% (Table 1). For treatment, seeds were evenly distributed in the bottom glass Petri dish and each seed lot was exposed to plasma for 60 s (CD60) or 120 s (CD120).

**Table 1:** Characteristics of plasma generation methods.

	Control (CON)	Plasma processed air (PPA)	Corona discharge (CD) reactor	DBD reactor in argon (ArDBD)	DBD reactor in air (airDBD)
Type of discharge	–	Microwave discharge	Corona discharge	Dielectric barrier discharge	Dielectric barrier discharge
Type of excitation	–	Microwave-driven	AC-driven	AC-driven	AC-driven
Frequency	–	2.45 GHz	10 kHz	10 kHz	19 kHz
Electric parameters	–	Consumed power: 1.1 kW	Voltage: 24 kV p-p with 50% duty cycle	Voltage: 12 kV p-p with 30% duty cycle	Voltage: 20 kV p-p
Gas used	Open air	Compressed air	Argon	Argon	Open air
Gas flow rate	–	73 slm	4 slm	2 slm	–
Treatment times	–	600 s	60 and 120 s	120 s	6 s
Average temperature at the seed coat (for maximum treatment time)	Ambient	23.0 ± 0.2 °C	42.9 ± 1.5 °C	32.6 ± 1.2 °C	29.8 ± 1.2 °C

#### Dielectric barrier discharge reactor with argon as working gas (ArDBD).

The ArDBD reactor consisted of a coaxial DBD made of a glass cylinder serving as dielectric mounted on top of a vortex mixer. The high voltage copper electrode was wrapped in spiral shape around the glass cylinder. The grounded electrode consisted of a copper rod introduced in its central axis. The detailed configuration of the laboratory scale ArDBD reactor (Fig 1B) is described in [45]. Argon flow rate was kept constant at 2 slm and applied voltage at 12 kV p-p with voltage duty cycle of 30% and frequency of 10 kHz (Table 1). Under these conditions, a filamentary discharge evenly distributed through the cylinder volume was produced. An image of the operating reactor is shown in S5 Fig in S1 File. Seeds were distributed between the electrodes for treatment. The vortex mixer was used at medium speed, allowing a constant mixing of the seeds. To ensure treatment reproducibility, the reactor was always operated with the same number of seeds, and the shaking speed was kept constant for all treatments. Each set of accessions was treated for 120 s.

#### Dielectric barrier discharge reactor with air as working gas (airDBD).

The airDBD reactor consisted of a rotating electrode assembled on a conveyor belt in modular configuration (Fig 1C). The high voltage rotating electrode was a copper cylinder covered with glass placed at 3 mm on top of the belt, while the bulk metal part below was grounded. Seeds were fed into the reactor by a funnel-like module, which homogeneously distributed the seeds on the belt and prevented them from stacking up. Therefore, one uniform layer of seeds prior to plasma treatment was created. When the seeds reached the electrode module, they acted as a counter electrode and the gap was reduced. By this, a discharge was ignited in open air on top of the seed surface (S5 Fig in S1 File). For the seed treatment, the reactor operated with 19 kHz and an applied voltage of 20 kV p-p (Table 1). The belt speed was kept at 8.3 mm s^-1^, which led to a treatment of the seeds for 6 s. After exposure to plasma, seeds were transported by the belt to a collecting container module.

#### Plasma processed air (PPA).

For indirect treatment, a microwave driven discharge was applied to produce PPA. Plasma was operated at a gas temperature of ~4000 K. For further details on the construction and composition of the device, see [46]. The flow rate of the compressed air was 73 slm (standard litre per minute), the incubation time 10 min. The exhaust PPA was cooled down to room temperature (about 23 °C, Table 1) before entering one litre glass incubation bottles with seeds. The PPA introduced in the bottles presented a brownish colour (S5 Fig in S1 File) that indicates formation of NO_2_ after plasma treatment. To stop the exposure of seeds to PPA, the incubation bottles were refilled twice with untreated compressed air.

### Germination experiments

To examine the effects of CAPP on germination of red clover, CAPP treated seeds were tested under controlled conditions in a germination cabinet (GC11, Flohr Instruments, The Netherlands). Germination tests took place directly after plasma treatment (8−14 days) as well as after around seven and a half months of storage at −5 °C (Batch 1: 228 days, Batch 2: 221 days, Batch 3: 230 days). An additional germination test was conducted in seedling trays under greenhouse conditions directly after plasma treatment. Each seed lot was tested with three replicates for all five CAPP treatments and compared to an untreated control. Each replicate consisted of 50 seeds. Batch 1 was tested in April and November 2021, Batch 2 end of May 2021 and beginning of January 2022, and Batch 3 end of July 2021 and end of January 2022.

For testing under controlled conditions, the seeds were sown on moisture-retaining filter paper and placed in the germination cabinet (Flohr Instruments, Nieuwegein, Netherlands). Conditions were set to automatic control of moisture, light and temperature (light for 11 h at 22 °C, darkness for 13 h at 15 °C). For testing under greenhouse conditions, two seeds per pot were sown into 25 planting pots (49 mm diameter) filled with organic turf material (Pikiersubstrat fein, Einheitserdewerk Uetersen, Werner Tantau, Germany). The intended temperature was 20 °C after sunrise and 15 °C after sunset. However, cooling was possible only by opening the windows. Germinated normal seedlings were counted after four, seven and eleven days after sowing (DAS). A normal seedling was defined as having a non-broken radicle with a length of at least 0.5 cm (visible only in germination chamber), an at least 0.5 cm long hypocotyl, and two (or in exceptional cases three) non-broken, fully developed cotyledons. For the experiment under controlled conditions, germinated normal seedlings were removed from the filter paper during counting. An example of germination and seedling growth is shown in S4 Fig in S1 File. Abnormal seedlings were determined after eleven days. For the calculation of germination percentage, only normal seedlings were counted.

### Pot experiment

Per seed lot, three seedlings from the germination experiment in the greenhouse were transferred into one pot (7 L; ED73 substrate, Einheitserdewerk Uetersen, Werner Tantau, Germany). They were cultivated under greenhouse conditions in three replicates for 40 days (Batch 1: 7^th^ May – 15^th^/16^th^ June 2021, Batch 2: 24^th^/25^th^ June – 3^rd^/4^th^ August 2021) or 53 days (Batch 3: 26^th^/27^th^ August – 18^th^/19^th^ October 2021). Number of shoots and shoot length per plant, as well as shoot fresh and dry matter (oven dried at 60 °C for two days) per pot were measured.

### Determination of seed colour

Seed colour was chosen as a proxy for seed coat pigments and/or other phenolic compounds and was correlated to normalized treatment effects of CAPP on germination. For standardized and comparable colour measurements, a visual analyser (electronic eye) equipped with a 25 mm lens (IRIS VA400, Winopal, Elze, Germany) was used. It was calibrated daily or before each series of measurements using an associated colour palette (colour reference ColorChecker, Winopal Elze, Germany). The electronic eye uses a chroma meter measure in the CIELAB L*a*b* colour system. The L*a*b* colour system consists of a lightness component (L*, 0 =* *black and +100 = white) and two chromatic components: the a* value represents green (−100 complete greenness) to red (+100 complete redness) while the b* value represents blue (−100 complete blueness) to yellow (+100 complete yellowness). Three sets with 50 seeds each (150 seeds in total) were used per seed lot of Batch 1 to determine the average colour (S1 Fig in S1 File). The images obtained by the visual analyser were cleaned from the background by a filter method after the capture to analyse only the colours of the seed. Image acquisition and calculations of the CIELAB colour space were done using the AlphaMOS software (Alpha M.O.S., France).

### Statistical analyses

We calculated treatment effects on seed germination for each accession at 4, 7, and 11 days after sowing (DAS). For each time point, we divided the mean cumulative percentage of normal seedlings (three replicates) after each plasma treatment by the corresponding value for the untreated control, and multiplied by 100. In other words, treatment effect (%) = 100 × [treated mean/ control mean].

Statistical analyses were performed using either R 4.4.0 [47] in the RStudio development environment [48] or Sigma Plot 13 (Systat Software, San Jose, California, USA). For germination percentages, a generalized linear model with the two factors treatment and accession was fitted (family = binomial with cumulative number of normal seedlings as success and others as failure) and subjected to an analysis of variance (“car” package; [49]) using R. For biomass, a linear model with treatment and accession was fitted and subjected to an analysis of variance (function “aov”). For number of shoots and shoot length, a linear mixed effects model with treatment and accession as fixed factor and pot as random factor was fitted using “lmer” in package “lme4” [50]. If treatment was significant, differences of each plasma treatment to the control were tested using the Dunnett test within the function “glht”. Spearman correlations were calculated using “cor.test”, and regressions were calculated using a linear model. Figures were created with the help of the R package “ggplot2” [51].

CIELAB colour space parameters L*, a* and b* were correlated to the treatment effect at each DAS using Spearman’s correlation in SigmaPlot 13 (Systat Software, San Jose, USA).

## Results

### Effect of plasma treatment on germination in a climate cabinet

**Fig 2:**
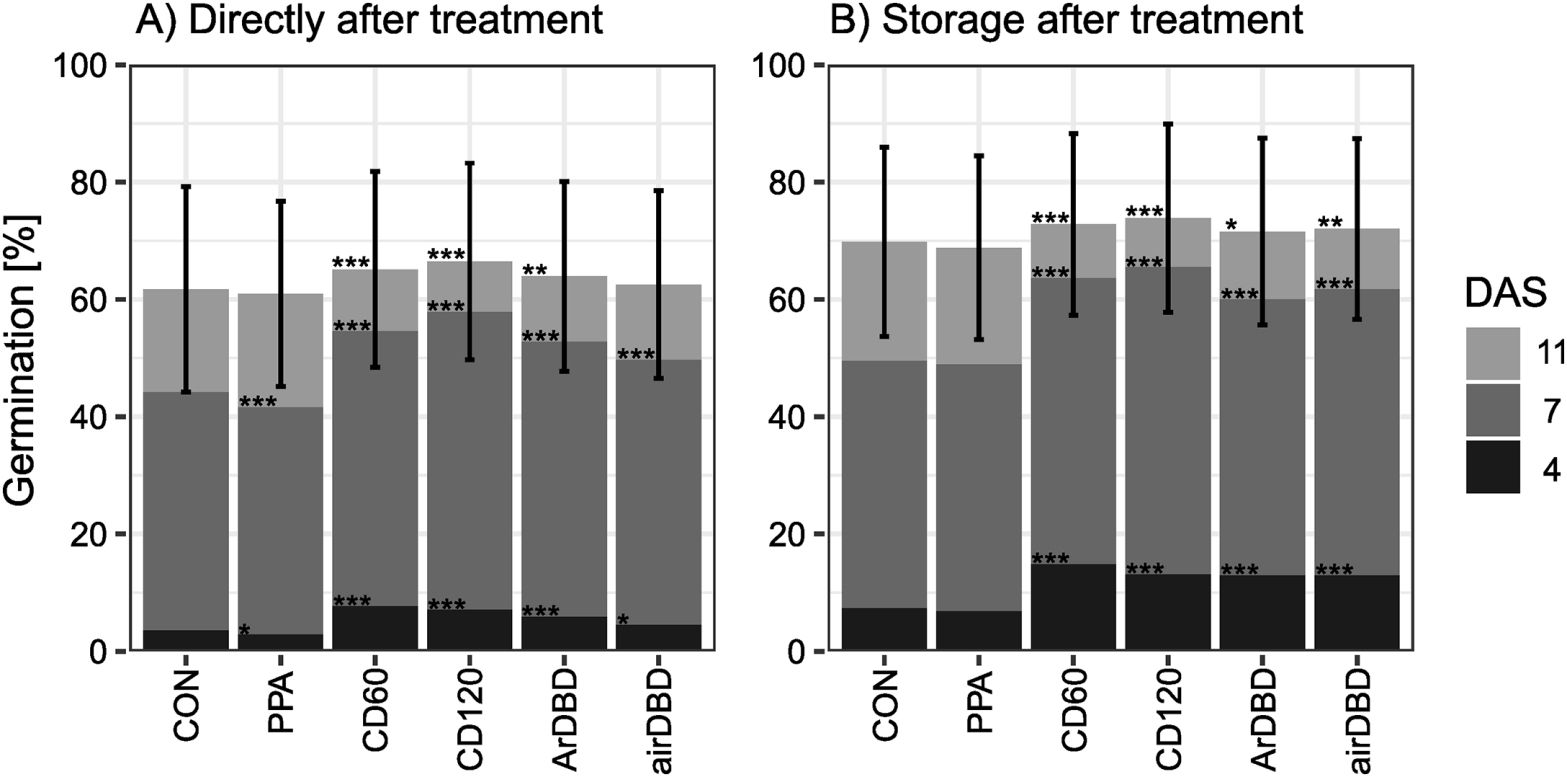
Germination percentage across all 60 seed lots. Directly after plasma treatment (A) and after storage (B) under controlled conditions after four, seven and eleven days after sowing (DAS). Mean ± SD for cumulative percentage on day eleven, n = 60 × 3 replicates. Asterisks indicate significant differences to control (p = 0.05 > * < 0.01 > ** < 0.001 > ***). For abbreviations, see text.

Averaged over 60 different seed lots of red clover, 61.7% of seedlings germinated normally under control conditions directly after plasma treatment, ranging from 23% (LE 1939_2017) to 89% (LE1593_2014), depending on seed lot. After storage, 69.8% of seedlings germinated normally under control conditions, from 39% (LE1939_2017) to 90% (LE1258_2015 and LE2807_2015; Fig 2, S2 and S3 Tables in S2 File). Harvest year had no effect on germination percentage in control groups (p = 0.065, not shown).

Seed treatment with CD60, CD120 and ArDBD resulted in a significantly higher number of normal seedlings (65.1%, 66.5% and 63.9%, resp.) than in the control groups directly after plasma treatment (Fig 2A). Increased germination percentages were shown for those and for airDBD also after storage (72.8%, 73.9%, 71.6% and 72.0%, resp.; Fig 2B). The germination percentages after four and seven days directly after plasma treatment and after storage were significantly increased for CD60 (7.7%, 54.6% directly; 14.8%, 63.6% after storage, resp.), CD120 (7.1%, 57.8% directly; 13.1%, 65.5% after storage), ArDBD (5.9%, 52.8% directly; 12.9%, 60.0% after storage) and airDBD (4.5%, 50.0% directly, 13.0%, 61.7% after storage) treated seeds, indicating a faster seedling emergence after direct plasma treatment than for control groups (3.8%, 44.2% directly, 7.3%, 39.5% after storage) or indirect plasma treatments (2.9%, 41.6% directly, 6.8%, 48.9% after storage). Treatment with PPA decreased the germination percentage directly after plasma treatment significantly (60.9%) and showed no effect after storage (68.8%). Correlations of plasma effects based on accessions between directly after plasma treatment and after storage were highest at seven DAS (rho = 0.358, p < 0.001), although generally low (S2 Fig in S1 File).

**Fig 3:**
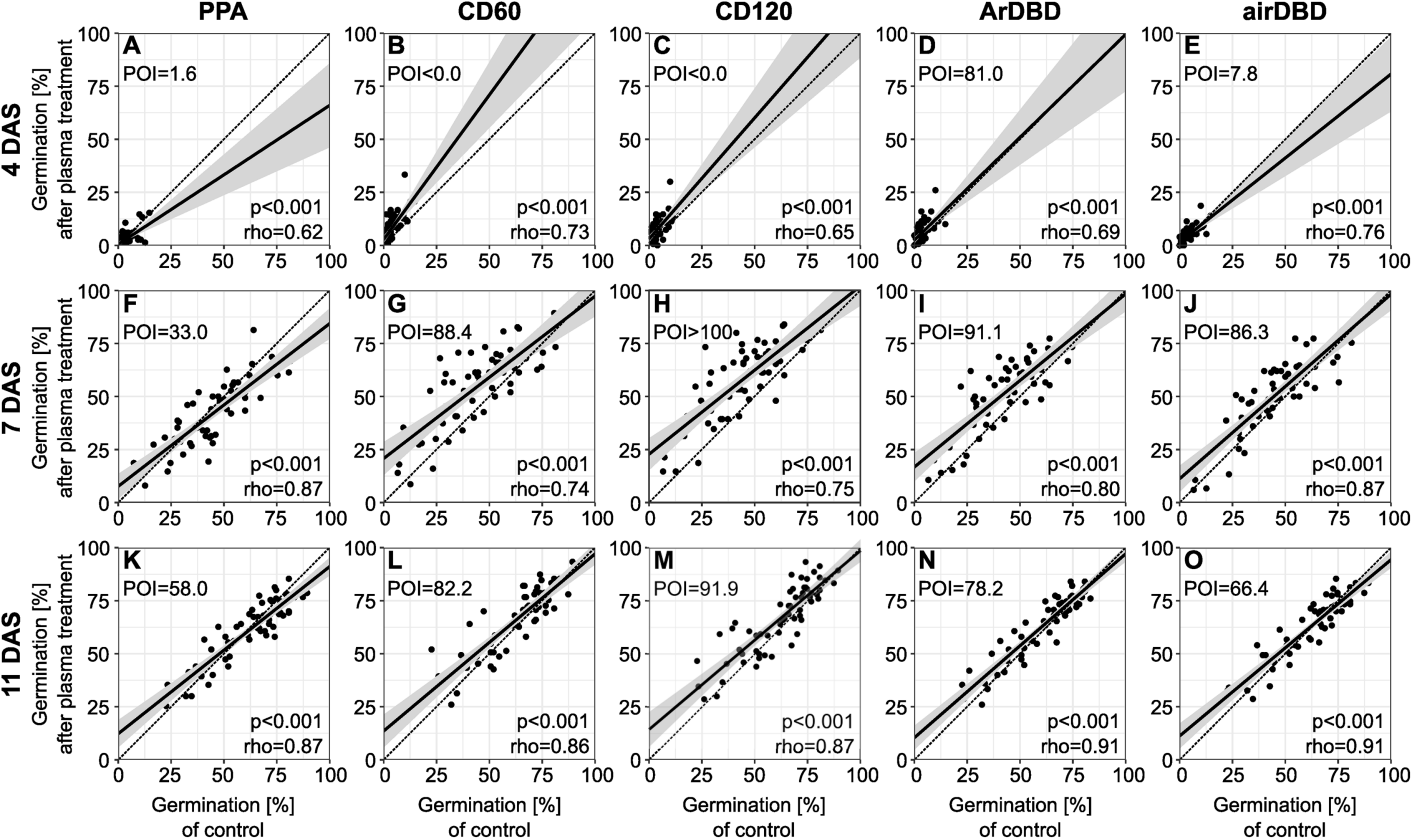
Relationship between germination percentage of control (x-axis) and plasma treatment (y-axis) for each observation time. Dashed line: no difference between control and treatment, bold line: regression line with 95% confidence interval. Given is point of intersection (POI) of both lines and Spearman correlation coefficient rho with p-value. n = 60 × 3 replicates. For abbreviations, see text.

The germination percentage directly after plasma treatment was positively correlated with the control on all days after sowing. Comparisons between the regression line and the identity line show the effect of plasma: With the control on the x-axis and the treatments on the y-axis, a positive plasma effect is shown by a regression line above the identity line. In agreement with the faster germination after all direct treatments (Fig 2), the plasma effect for most seed lots was positive at lower germination rates at seven DAS for all direct treatments (CD60, CD120, ArDBD, airDBD) (Fig 3). The positive effect was reduced at eleven DAS. The germination percentage, above which no positive effect can be expected, was lowest for PPA (33.0% germination under control at seven DAS and 58.0% at eleven DAS) and highest for CD120 (positive effect up to computational 100% germination under control at seven DAS and 91.9% at eleven DAS). However, the effect was highly dependent on seed lot with some seed lots showing a negative effect of plasma treatment (LE1725_2019, LE1540_1990b, LE1374_2012, LE2838_2018, LE1258_2015, S2 Table in S2 File), others with positive effects for all types of plasma treatment (e.g., LE1725_2018 as opposed to the same accession with harvest year 2019 - LE1725_2019 – with negative effects), but most with mixed effects (see S2 Table in S2 File).

Seed colour determined for accessions of Batch 1 revealed significant correlations with treatment effects (germination percentage of treated seed lots divided by germination percentage of control) across all three observation dates for PPA and CD120. Colour index b* (blue component) correlated significantly positive with effects of PPA treatment, whereas colour index L* (lightness) correlated significantly negative with effects of CD120 exposure (Table 2). This means that the effect of PPA treatment on seed germination was less negative in seeds with higher b* index (more yellow). Effects of CD120 on germination relative to control were more positive in darker seeds (lower L*). Germination at eleven DAS in control seeds was not correlated to colour indices.

**Table 2:** Spearman correlation between germination percentages and seed colour for Batch 1.

		a*	b*	CON 11 DAS	PPA effect	CD60 effect	CD120 effect	ArDBD effect	airDBD effect
	n	60	60	20	58	58	58	58	58
L*	rho	0.197	**0.866**	0.146	0.141	0.101	**−0.297**	−0.064	0.079
	*p*	0.131	**<0.001**	0.534	0.288	0.451	**0.024**	0.631	0.554
a*	rho		**0.364**	0.164	0.003	0.083	0.002	−0.003	−0.007
	*p*		**0.004**	0.484	0.980	0.533	0.986	0.979	0.961
b*	rho			0.064	**0.281**	0.186	−0.083	−0.004	0.103
	*p*			0.782	**0.033**	0.161	0.536	0.978	0.439

Control groups (eleven DAS) and CAPP effects were correlated with seed colour (all averaged over three replicates). P-values ≤ 0.05 are indicated in bold. n: number of samples, rho: correlation coefficient. L*: lightness; a*: green-red; b*: yellow-blue. Values for two seed lots with zero germination in control at four DAS were omitted from the analysis. For abbreviations, see text.

### Effect of plasma treatment on germination and plant development in the greenhouse

**Fig 4:**
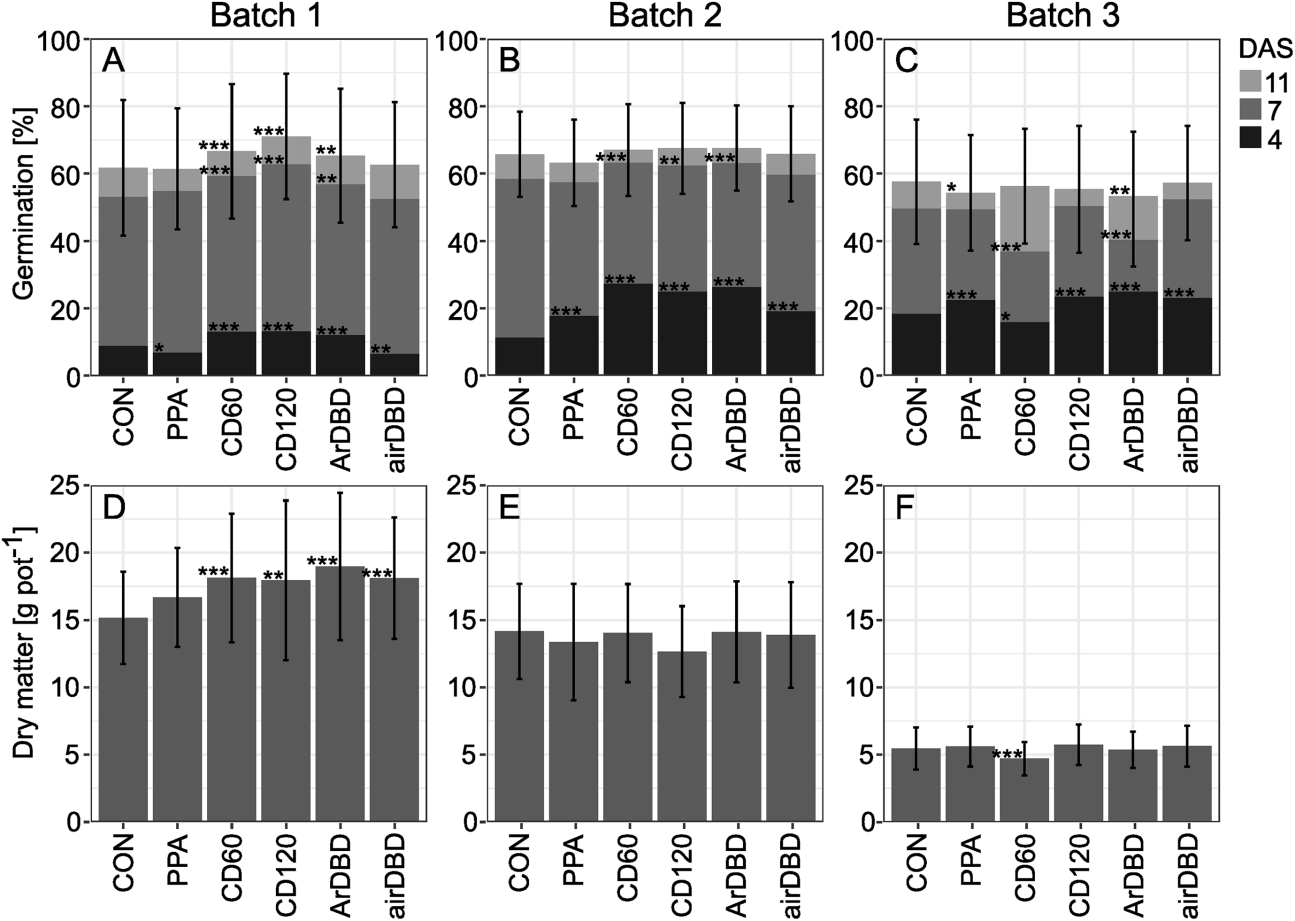
Germination percentages and biomass of red clover under greenhouse conditions. Germination percentage four, seven and eleven days after sowing (DAS) in potting substrate (A-C) and shoot dry matter (D-F) of young red clover plants. Mean ± SD (A-C: only for cumulative percentage on day 11), n = 20 accessions per Batch with 3 replicates. Asterisks indicate significant differences to control (p = 0.05 > * < 0.01 > ** < 0.001 > ***). For abbreviations, see text.

Contrary to the conditions in the germination cabinet, the conditions in the greenhouse varied between the three tested batches of seed lots with shorter days and lower light intensity for Batch 3 in September. Therefore, they were evaluated separately. Germination percentage under greenhouse conditions showed a similar pattern for Batch 1 compared to germination cabinet experiments with significantly higher germination percentages after CD60, CD120 and ArDBD treatments compared to control after all evaluated time points (Fig 4A). In Batch 2, all plasma treatments resulted in higher germination percentages after four days, but in slight increases after seven days only for CD60, CD120 and ArDBD (Fig 4B). Batch 3 had significantly lower germination percentages after seven days for CD60 and ArDBD compared to the control, and after eleven days for PPA and airDBD (Fig 4C). Overall, germination percentages after four and seven days were higher for plants cultivated in the greenhouse in contrast to germination tests in the climate cabinet.

Biomass of young plants was significantly higher after all direct plasma treatments only in Batch 1 (on average 18.3 g compared with 15.2 g in control; Fig 4D), but lower after CD60 treatment in Batch 3 (Fig 4F). Except for CD120, maximum shoot length per plant was higher after plasma treatment in Batch 1 (on average 443 mm instead of 386 mm in control). Lower values for shoot length and number of shoots were found after CD60 treatment in Batch 3 (S3 Fig in S1 File). Number of shoots was not affected by plasma treatment in Batches 1 and 2 (S3 Fig in S1 File).

## Discussion

### Direct CAPP treatment increases red clover germination

Plasma agrotechnology is an upcoming field with promising results for increasing plant growth and reducing pesticide use, that still has to overcome some challenges before an integration into agricultural practices is feasible [52]. Plasma, often described as an ionized gas, produces reactive species that interact with the seed surface. These interactions can increase seed permeability, allowing for better water absorption and gas exchange, which is an essential step in the germination process. Additionally, plasma can stimulate internal biochemical pathways, leading to improved seedling growth. In this way, plasma treatment serves as a physical and chemical stimulus enhancing overall seed germination. The goal of this study was to test the effect of seed treatment with different plasma treatment systems on the germination of red clover accessions maintained at the IPK Gene Bank directly after plasma treatment in the germination chamber and under greenhouse conditions. Furthermore, it was tested whether storage after plasma treatment had further influence on germination capacity. In our study, all direct plasma treatments resulted in a significantly accelerated germination, but the measured maximum germination percentage was only slightly increased (highest after CD120 treatment compared to control: 66.5%/61.7% (directly), 73.9%/69.8% (storage), 64.6%/61.7% (greenhouse), respectively). The overall trends of the plasma effect were the same in all germination experiments, i.e., directly and storage after treatment under controlled conditions and germination on soil in the greenhouse. The maximum number of germinated normal seedlings was slightly higher after storage for all treatments and control. A natural reduction of hardseededness within these seven months is unlikely due to the much longer storage period before plasma treatment. However, an environmental influence on the conditions in the germination cabinet cannot be completely excluded and is certain in the greenhouse with respect to light-dark and temperature modulation as well as humidity.

The germination in soil under greenhouse conditions was in general faster compared to germination on filter paper. Environmental conditions [53] as well as soil type and texture [54] can have an effect on germination time.

In our study, CD120 treatment resulted in the highest increases in germination percentages irrespectively of germination conditions (climate cabinet versus greenhouse), especially after seven and eleven days. An increase in germination speed and maximum germination percentage was frequently observed for various plant species [38,55,56]. These effects were either attributed to chemical modifications in the seed coat composition [39,41] and associated wettability or to the physiological effect of RONS exposed to the seeds during CAPP treatment [57]. Elevated wettability of seed coats after direct CAPP exposure occurs due to oxidation, nitration and nitrosation reactions by reactive species in the gas phase. Several studies indicate a reversible attachment of oxygen-containing functional groups [39,41,58,59] or lipid peroxidation [60], which ultimately results in the observed change in water uptake and accelerated germination. Moreover, erosion, cracking and/or etching events are discussed for advanced water uptake and following germination as well [58,59,61,62]. Indirect plasma treatments like PPA do not induce changes in the seed coat wettability [46,63,64] and has no positive effect on germination in our study as well.

On the other hand, RONS produced during plasma generation can act as signalling molecules and influence hormone balances inside of seeds [34]. Stimulated germination was associated with an increase in gibberellin (GA) concentration, which is known to promote germination [65,66]. During physiological dormancy, the ratio between GA and its antagonist abscisic acid (ABA) plays an important role, with dormancy continuing when ABA levels are high while concentration of GA is low [67]. This natural physiological dormancy is released during storage along with an increase in the GA/ABA ratio [68]. In addition, ethylene also acts as an antagonist of ABA and stimulates germination [67]. Next to affecting the hormone status, plasma treatment can have an influence on the antioxidant system along with enzyme activity. An enrichment of intracellular RONS was observed by [69] after low doses of CAPP. These RONS enhanced the level of intracellular antioxidants and antioxidant enzymes, and ultimately stimulated growth of *Arabidopsis thaliana* seedlings.

Next to studies showing stimulating effects of CAPP, also neutral or negative effects are published as reviewed by [70]. Direct comparisons between studies are often difficult because plasma discharges display a high variability and devices are mostly self-built along with treatment and operational procedures which are not standardized [56]. In general, effects on seed germination were shown to be variable and depending on the plasma device [31]. Inhibitory effects of plasma treatments, as observed in our study for indirect plasma treatment using PPA, can likely be attributed to high levels of radicals and reactive species within plasma such as nitric oxides (NO_x_). Negative effects on asparagus seed germination were observed using oxygen as feed gas [71], on hemp seeds after indirect plasma treatment using downstream microwave plasma apparatus and a mixture of oxygen and argon as feed gas [72], on spinach after long-term treatment using high-voltage nanosecond pulsed plasma [65] and on brown rice after DBD treatment longer than 100 s [73]. However, since the number of abnormal seedlings was unaffected by plasma (S2-S4 Tables in S2 File), degradation of DNA by the tested plasma treatments is unlikely.

### CAPP effects on red clover germination are seed lot-specific

Accessions for this study were selected in order to represent a high range of initial maximum germination percentages, which resulted in having seed lots of different accessions from different harvest years. A greater effect of direct plasma treatment was found for seeds with initially low germination percentages. This could be due to the finite nature of percentages, i.e., low percentages have low margin to become even lower and high percentages to become even higher. Additionally, other dormancies or disorders might occur in red clover such as physiological dormancies, seed coat thickness or low embryo vitality that might be unaffected by plasma treatment. Overall, seed treatment, being biological, chemical or physical, can remove reversible barriers (dormancy, mild pathogen load, poor imbibition) but cannot restore non‑viable seeds. The viable fraction of seeds, as well as the dormancy complexity can be responsible for the remaining non-germinating seeds in our study. A small percentage of seeds produced abnormal seedlings in our study, which were not shown here, but are presented in the S2-S4 Tables in S2 File. Percentage of abnormal seedlings were similar in control and treated samples. We were not able to distinguish between dormant and non-viable seeds among the non-germinated seeds.

Irreversible damage during maturation, handling, or storage can reduce seed vigour as these lesions might be only partly reversible. More complexity arises when considering mixed maturity, moisture histories, and genetic backgrounds which can create subpopulations and lot heterogeneity.

During storage, seeds lose their viability with genotypic and environmental effects on the rate of the loss, e.g., shown by [74] for barley. The seed lots in our study were from different years. Environmental conditions during seed development and processing operations (harvest, threshing) can have an influence on seed quality [6,33]. For example, the year 2017 (eight seed lots date back to that year) was a year with high precipitation. Severe moisture stress can increase the percentage of hard seeds [75]. Additionally, reactions to such abiotic influences can depend on the genetic background. For example, environmental conditions and the genetic background can have an influence on secondary metabolites and therefore seed colour. The overall impact of CAPP on germination of red clover was correlated to seed colour by [35], using sorting of seeds of one accession (Arimaiciai) visually into classes of yellow, brown and dark purple. The authors reported that germination of yellow seeds was stimulated stronger compared to dark purple and brown seeds and associated this to the content of pigments as well as other phenolic compounds. In brown and purple seeds stimulating CAPP induced changes in H_2_O_2_ generation were counterbalanced by higher amounts of ROS scavengers in the seed coat, compared to yellow seeds with less pigments. This is in line with our study, where we found a positive correlation between yellowness of seeds and the effect of PPA. However, the effect of CD120 treatment was negatively associated with brightness, i.e., more positive CAPP effects were detected in dark seeds. Overall, no correlation between maximum germination percentage and seed coat colour was found for control seeds, similar to the above-mentioned study, but adverse to previous reports for legumes [76,77]. However, we tested only Batch 1 as a pilot study for a proof-of-concept of using the method of CIELAB and correlations to plasma effects. In order to validate the correlations, subsequent studies should include determination of seed colour, complemented with seed coat pigments and secondary (phenolic) metabolites using probably at first accessions with uniform colour within one seed lot (S1 Fig in S1 File).

Additionally, seed water content was found to interact with DBD plasma treatment resulting in higher plasma effects for seeds with higher water content [78]. Usually, the seeds at IPK are stored with a water content of 5–8%, but deviations might occur and the water content of the seeds used for this study was not measured before plasma treatment.

Seed age was not correlated to plasma effects in our study. [33] found a stimulating effect for seeds of the red clover cultivar Vyčiai harvested only half or 1.5 years prior to the plasma treatment and not for 4.5 years old seeds with reduced germination. However, all our seeds were stored for more than 1.5 years in the gene bank before plasma treatment (S2 Table in S2 File).

In addition, seed treatment has to face a trade‑off between effectiveness and treatment injury: More aggressive treatments (e.g., chemical oxidants, plasma, intense scarification) might benefit some seeds but damage others, overall creating a plateau below the maximum attainable germination.

Altogether, to be fully able to maximize germination, the natural boundary of seed germination for a specific seed lot would have to be pre-defined. This could be done using X‑ray technologies or by applying tetrazolium viability tests to estimate the number of viable seeds.

### CAPP effect on seedling growth and biomass

While the germination percentage was similar between at least Batch 1 and 2 under greenhouse conditions, the biomass determined after 40 days (Batch 1 and 2) and 53 days (Batch 3) of growth differed greatly between the three independent batches. While in Batch 1, starting in May, a small positive effect of direct plasma treatment was observed regarding dry matter content, plasma treatment did not affect biomass in Batch 2 and 3, which were initiated in June and August. Growth conditions in the greenhouse aggravated with every batch (e.g., day length, temperature), which was visible in a decrease in overall biomass from Batch 1 to 3, resulting in stunted growth in Batch 3. Due to the lack of seed lots that were replicated in between all three batches, a differentiation between genotypic and the proposed environmental effect is not possible. Nevertheless, differences in day length and amount of daily photosynthetic active radiation were present.

Under optimal growth conditions, plasma displayed stimulating effects on plant growth in a number of different plant families analysed so far: Stimulation of seedling development and growth by DBD treatment depended on treatment time for tomato [79] and cucumber [80], and on the type of plasma and the cultivar in durum wheat [81]. Root growth in lentil but not in pea was promoted by DBD, while shoot dry matter was not affected [82]. Moreover, plant growth in greenhouse and field trials demonstrated stimulating CAPP effects on growth of two different cultivars of red clover [33,35], maize and wheat [83–86] (one cultivar/accession each). Physiological mechanisms behind these observed stimulations were connected to effects on primary metabolism, the activity of the cell internal antioxidant system [87] and internal cycling of RONS [69], the concentration and ratio of plant hormones (e.g., increase in hormones related to stress response [88]), changes in secondary metabolites [70] and elevated total content of isoflavonoids in the leaves and roots of red clover [33].

Additionally, plasma treatment displayed stimulation under adverse and stress conditions such as salt stress [89], drought stress [31,90–92], and biotic stresses [93 and references therein]. Increased stress tolerance after direct or indirect plasma treatment was associated to enhanced antioxidant activity, activation of signalling cascades associated with reactive oxygen and heat shock factors, increased accumulation of osmolytes and, in the case of biotic stress, activation of plant defence mechanisms [93 and references therein].

## Conclusion and future perspective

Direct CAPP treatment of red clover seeds displayed stimulating effects on seed germination averaged over 60 different seed lots (various accessions and harvest years). The magnitude of stimulation was dependent on accession and plasma device with corona discharge showing the strongest and most consistent positive effect. Overall, the initial germination percentage of 62% or 70%, depending on experiment, was not increased above 80% after CAPP treatment, a common threshold for storage in gene banks. Our study confirms that plasma effects on seeds are not consistent across different red clover accessions. Since the dependences are not fully understood, further research should elucidate the basis for the observed accession dependency by, e.g., analysing seed coat compounds in correlation to seed colour along with the effect of CAPP on the cell specific redox system.

Additionally, the limiting factor for germination should be determined, which might include different dormancy types, differences in seed vigour within the same lot or pathogen infection. It has to be confirmed that viability can actually exceed 80% before investing in treatment efforts. If physical dormancy has to be overcome, scarification along with CAPP might be successful. On the other hand, physiological dormancy might be alleviated by a combination of hydropriming, application of hormones, temperature gradients along with CAPP. Pathogen contamination can be reduced by chemical or biological seed dressing or using plasma- processed air along with direct CAPP. A combination of methods as well as additional plasma sources and operating parameters remain to be tested.

For an implementation of CAPP as future application in the agricultural sector, several considerations must be addressed including scalability, standardization of treatment protocols and the need for a more comprehensive mechanistic understanding. The scalability should be tailored to the target plant species and the typical quantity of seeds available for treatment (e.g., agricultural products vs. medicinal plants). While PPA is already capable of treating kilograms of seeds within minutes, a corona discharge reactor can only process some grams. Therefore, scalability is a significant limitation for CAPP treatments based on CD or DBD due to restrictions in treatment area, device complexity, uniformity of treatment, sample geometry and size. In contrast, plasma processed air offers a practical and flexible solution for treating larger volumes of biological products such as seeds, enabling broader adoption in industrial and agricultural applications. However, efficacy and the specific nature of reactive species differ, so application requirements must be carefully considered when choosing the treatment modality. Additionally, energy requirements and costs have to be considered. Furthermore, there is a lack of standardized parameters for controlling and monitoring plasma performance with no universally accepted set of parameters applicable across different CAPP devices. Currently, parameters have to be adjusted for each plant species (or accession, as in our case) and desired effects. Addressing these challenges is essential for the transition from the test phase to a broader adoption of this technology.

## Supporting information

S1 File S1–S5 Figs. S1 Fig. Exemplary seed photos of all 20 seed lots used for determination of seed colour parameters. Scale valid for all. S2 Fig. Correlations between the plasma effect directly after plasma treatment and six months after plasma treatment. Plasma effect was calculated as percentage of untreated control (percentage of normal seedlings for 60 seed lots) at 4, 7 and 11 days after sowing (DAS) under controlled conditions in a germination cabinet. PPA: plasma processed air, CD60/120: corona discharge for 60/120 s, ArDBD/airDBD: argon/air dielectric barrier discharge. p-value and Spearman correlation coefficient rho are displayed. S3 Fig. Development of red clover plants under greenhouse conditions in potting substrate: Fresh matter (A-C), number of shoots (D-F) and maximum shoot length (G-I) of young red clover plants (three plants per pot) without or with seed treatments with plasma after 40 days for Batch 1 and 2 and 53 days for Batch 3 (CON: control, PPA: plasma processed air, CD60/120: corona discharge for 60/120 s, ArDBD/airDBD: argon/air dielectric barrier discharge). Mean value and SD are shown (A-C: only for cumulative percentage on day 11), n = 20 accessions per Batch with 3 replicates. Asterisks indicate significant differences to control (p = 0.05 > * < 0.01 > ** < 0.001 > ***). S4 Fig. Red clover seedlings of accession LE 1376/2019 as example of seedling growth during germination tests under controlled conditions and in the greenhouse (accession is sown with two seeds in each of the planting holes of the four right columns and the left uppermost planting hole). S5 Fig. Generated discharge for each of the direct treatment plasma sources and treatment bottle filled with plasma processed air (with brownish colour). a) PPA: plasma processed air, b) CD: corona discharge, c) ArDBD: argon dielectric barrier discharge, d) airDBD: air dielectric barrier discharge.
(PDF)

S2 File S1–S5 Tables. S1 Table. Information about accessions, accessed via the Genebank Information System at gbis.ipk-gatersleben.de. S2 Table. Number of normal seedlings (out of 50) counted 4, 7 and 11 days after sowing (DAS) and of abnormal seedlings counted 11 DAS under controlled conditions in a germination cabinet without or with seed treatments with plasma after short-term storage (CON: control, PPA: plasma processed air, CD60/120: corona discharge for 60/120 s, ArDBD/airDBD: argon/air dielectric barrier discharge). Mean value and SD for 3 replicates. S3 Table. Number of normal seedlings (out of 50) counted 4, 7 and 11 days after sowing (DAS) and of abnormal seedlings counted 11 DAS under controlled conditions in a germination cabinet without or with seed treatments with plasma after 6-months storage (CON: control, PPA: plasma processed air, CD60/120: corona discharge for 60/120 s, ArDBD/airDBD: argon/air dielectric barrier discharge). Mean value and SD for 3 replicates. S4 Table. Number of normal seedlings (out of 50) counted 4, 7 and 11 days after sowing (DAS), number of abnormal seedlings counted 11 DAS under greenhouse conditions and plant parameters (shoot number (n), maximum shoot length (mm), fresh matter (g) and dry matter (g)) without or with seed treatments with plasma after short-term storage (CON: control, PPA: plasma processed air, CD60/120: corona discharge for 60/120 s, ArDBD/airDBD: argon/air dielectric barrier discharge). Mean value and SD for 3 replicates. S5 Table. CIELAB L*a*b* colour system indices for accessions of Batch 1. Mean value and SD for 3 replicates.
(XLSX)
