# Supplementary material for: Influence of cold atmospheric pressure plasma treatment on germination and plant biomass of Trifolium pratense L
Source: PLoS One. 2025 Sep 9;20(9):e0332166. doi: 10.1371/journal.pone.0332166 (PMC12419647; doi:10.1371/journal.pone.0332166)
Supplement: S1 File — S1–S5 Figs. S1 Fig. Exemplary seed photos of all 20 seed lots used for determination of seed colour parameters. Scale valid for all. S2 Fig. Correlations between the plasma effect directly after plasma treatment and six months after plasma treatment. Plasma effect was calculated as percentage of untreated control (percentage of normal seedlings for 60 seed lots) at 4, 7 and 11 days after sowing (DAS) under controlled conditions in a germination cabinet. PPA: plasma processed air, CD60/120: corona discharge for 60/120 s, ArDBD/airDBD: argon/air dielectric barrier discharge. p-value and Spearman correlation coefficient rho are displayed. S3 Fig. Development of red clover plants under greenhouse conditions in potting substrate: Fresh matter (A-C), number of shoots (D-F) and maximum shoot length (G-I) of young red clover plants (three plants per pot) without or with seed treatments with plasma after 40 days for Batch 1 and 2 and 53 days for Batch 3 (CON: control, PPA: plasma processed air, CD60/120: corona discharge for 60/120 s, ArDBD/airDBD: argon/air dielectric barrier discharge). Mean value and SD are shown (A-C: only for cumulative percentage on day 11), n = 20 accessions per Batch with 3 replicates. Asterisks indicate significant differences to control (p = 0.05 > * < 0.01 > ** < 0.001 > ***). S4 Fig. Red clover seedlings of accession LE 1376/2019 as example of seedling growth during germination tests under controlled conditions and in the greenhouse (accession is sown with two seeds in each of the planting holes of the four right columns and the left uppermost planting hole). S5 Fig. Generated discharge for each of the direct treatment plasma sources and treatment bottle filled with plasma processed air (with brownish colour). a) PPA: plasma processed air, b) CD: corona discharge, c) ArDBD: argon dielectric barrier discharge, d) airDBD: air dielectric barrier discharge. (PDF) [file pone.0332166.s001.pdf]

Supplemental material

to

Kavka et al.

Influence of cold atmospheric plasma treatment

on germination and plant biomass of *Trifolium pratense* L.

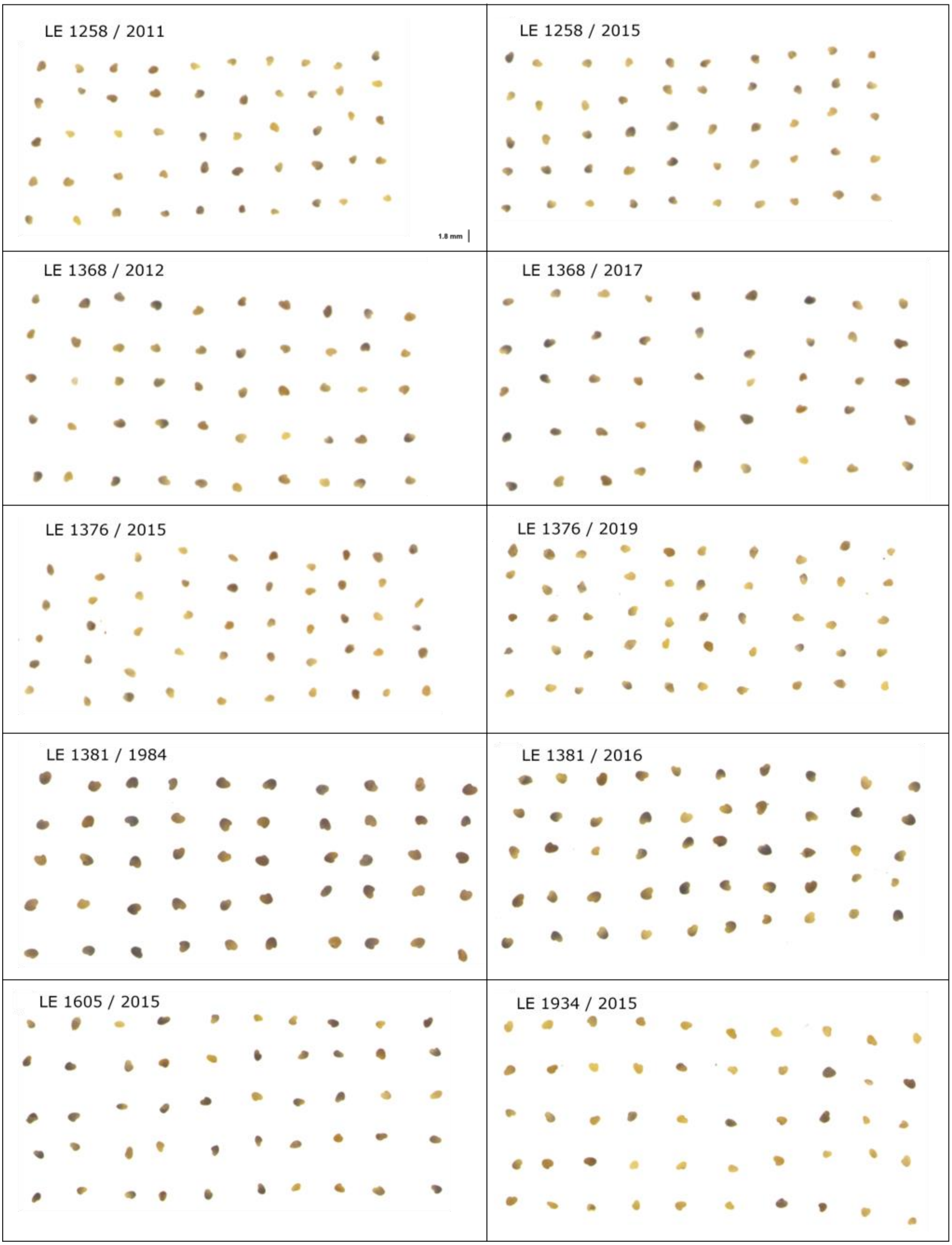

Supplemental Figure S1, first part. **Exemplary seed photos of all 20 seed lots used for determination of seed colour parameters.** Scale valid for all.

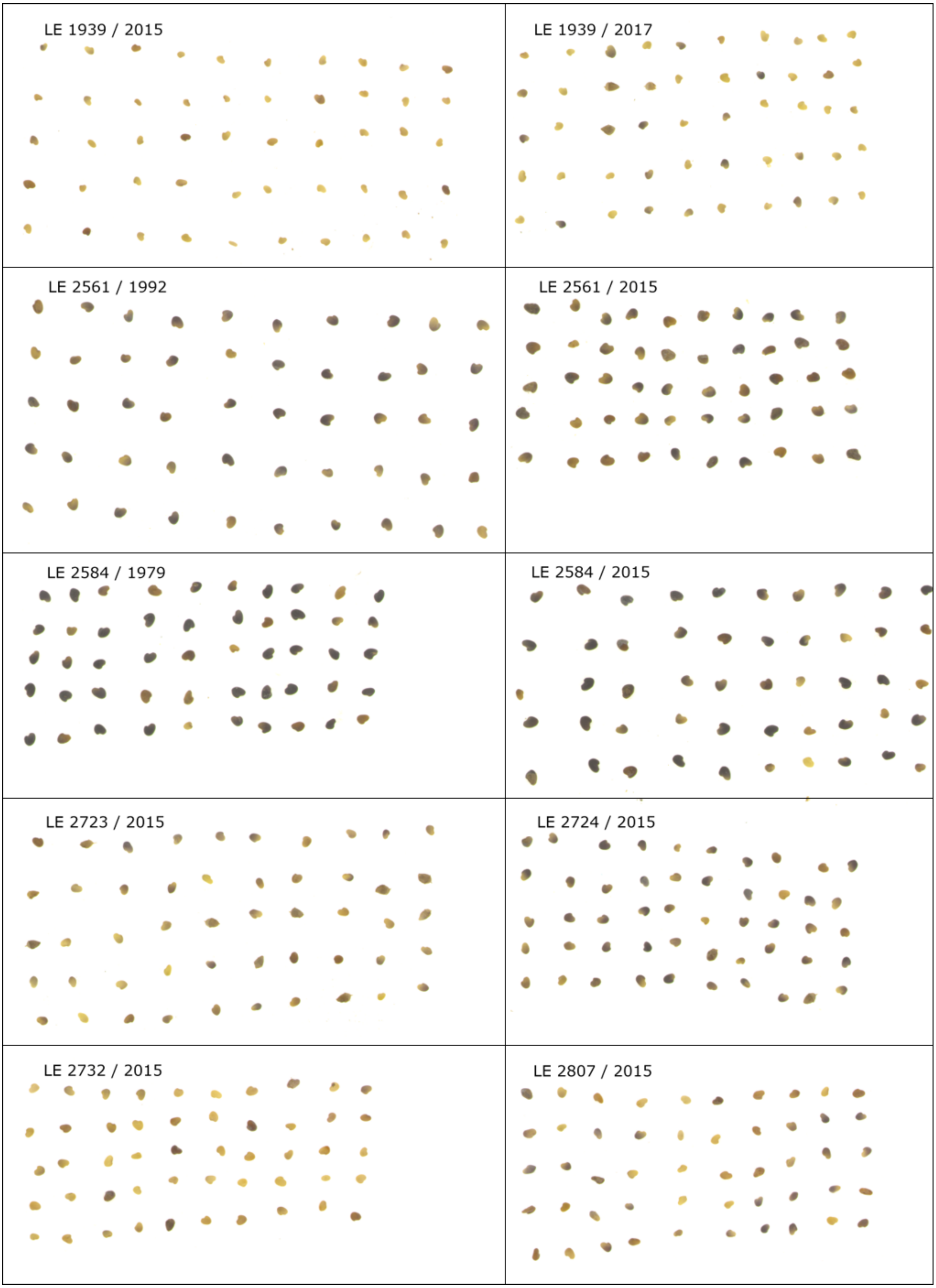

Supplemental Figure S1, second part: **Exemplary seed photos of all 20 seed lots used for determination of seed colour parameters.** Scale valid for all.

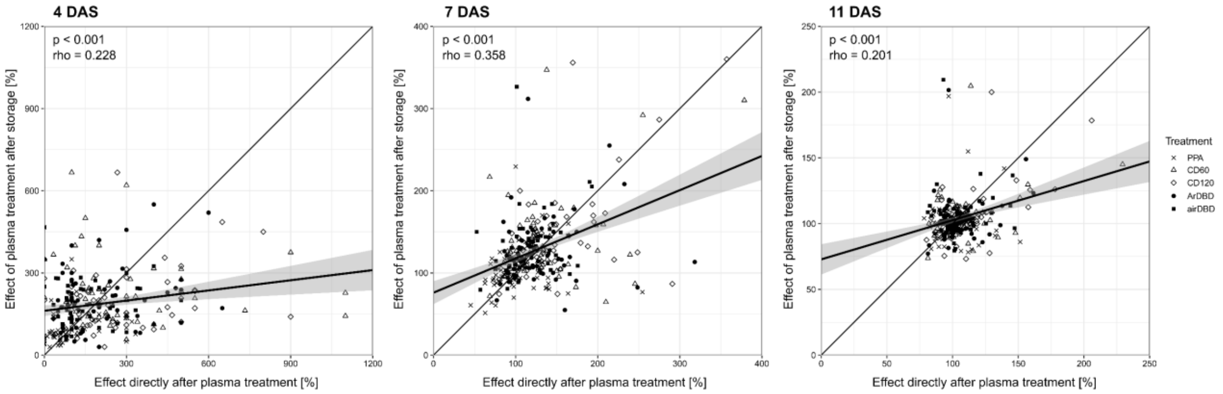

Supplemental Figure S2: Correlations between the plasma effect directly after plasma treatment and six months after plasma treatment. Plasma effect was calculated as percentage of untreated control (percentage of normal seedlings for 60 seed lots) at 4, 7 and 11 days after sowing (DAS) under controlled conditions in a germination cabinet. PPA: plasma processed air, CD60/120: corona discharge for 60/120 s, ArDBD/airDBD: argon/air dielectric barrier discharge. p-value and Spearman correlation coefficient rho are displayed.

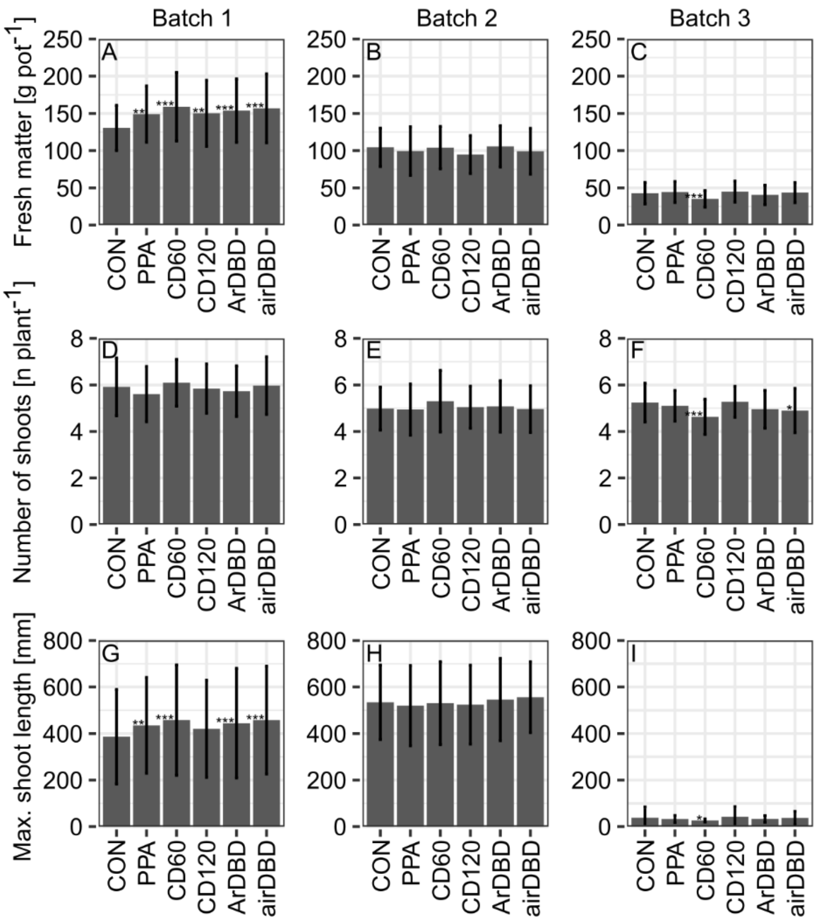

Supplemental Figure S3: Development of red clover plants under greenhouse conditions in potting substrate: Fresh matter (A-C), number of shoots (D-F) and maximum shoot length (G-I) of young red clover plants (three plants per pot) without or with seed treatments with plasma after 40 days for Batch 1 and 2 and 53 days for Batch 3 (CON: control, PPA: plasma processed air, CD60/120: corona discharge for 60/120 s, ArDBD/airDBD: argon/air dielectric barrier discharge). Mean value and SD are shown (A-C: only for cumulative percentage on day 11), n=20 accessions per Batch with 3 replicates. Asterisks indicate significant differences to control (p=0.05>*<0.01>**<0.001>***).

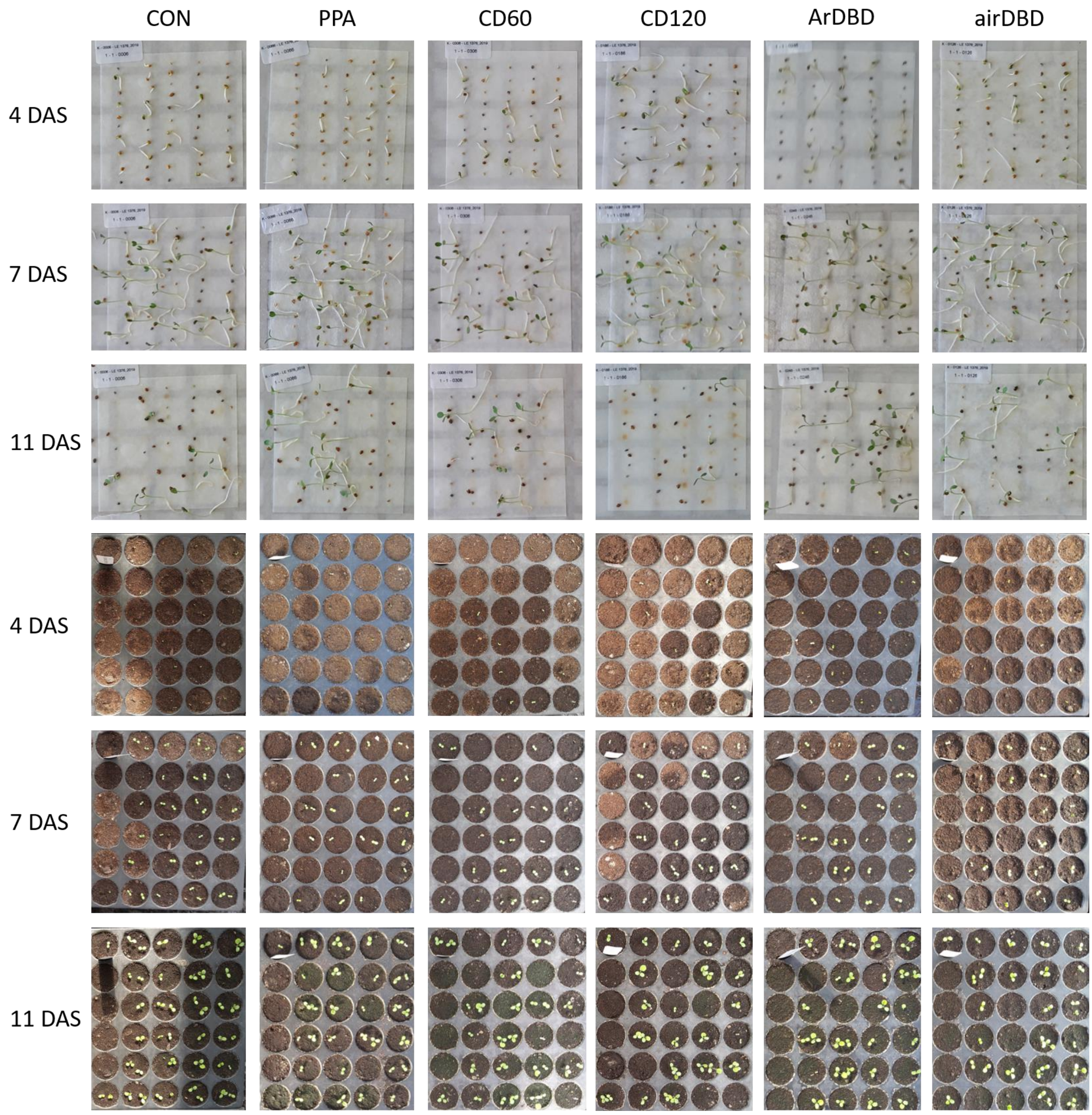

Supplemental Figure S4: Red clover seedlings of accession LE 1376/2019 as example of seedling growth during germination tests under controlled conditions and in the greenhouse (accession is sown with two seeds in each of the planting holes of the four right columns and the left uppermost planting hole) after plasma treatment of the seeds (CON: control, PPA: plasma processed air, CD60/120: corona discharge for 60/120 s, ArDBD/airDBD: argon/air dielectric barrier discharge).

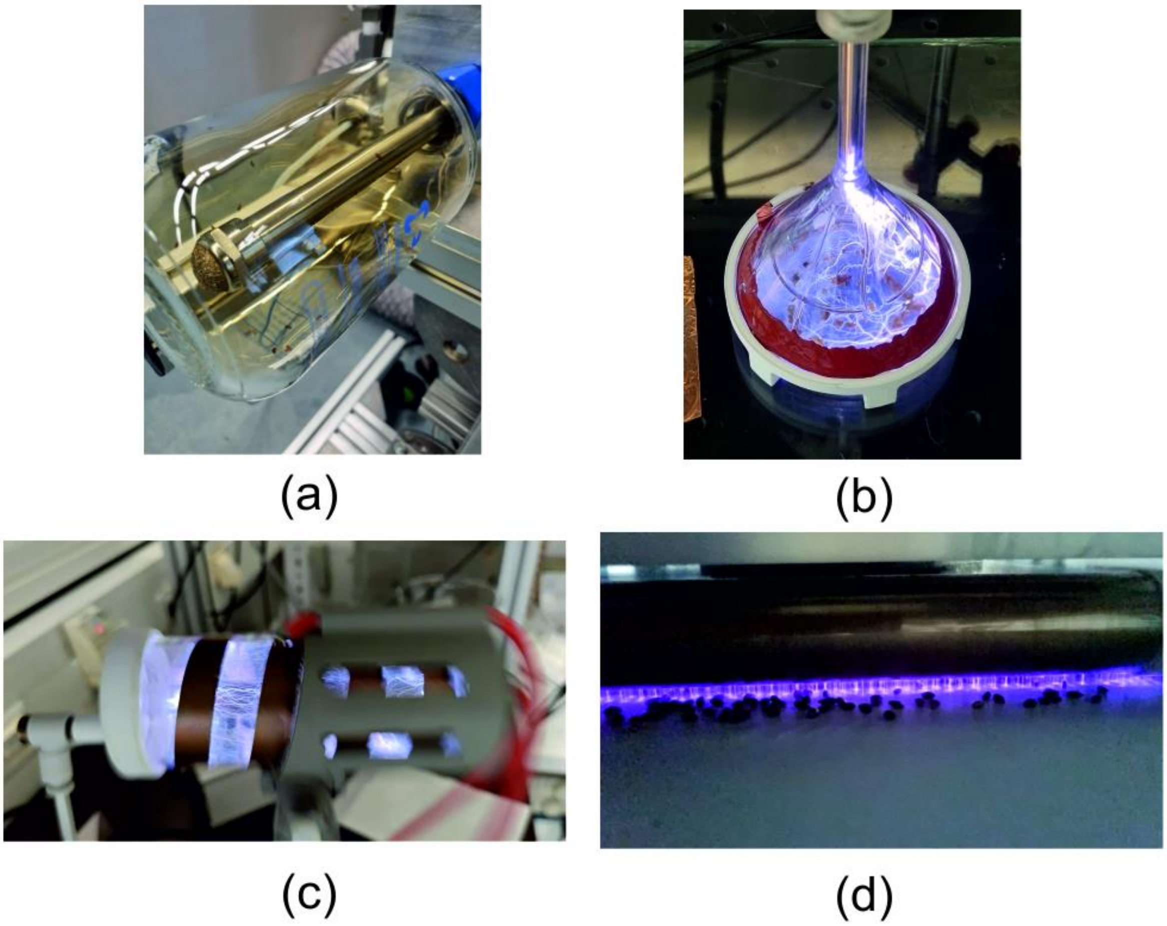

Supplemental Figure S5: Generated discharge for each of the direct treatment plasma sources and treatment bottle filled with plasma processed air (with brownish colour). a) PPA: plasma processed air, b) CD: corona discharge, c) ArDBD: argon dielectric barrier discharge, d) airDBD: air dielectric barrier discharge.
